# The effect of social categorization on trust decisions in a trust game paradigm

**DOI:** 10.3389/fpsyg.2015.01568

**Published:** 2015-10-12

**Authors:** Elena Cañadas, Rosa Rodríguez-Bailón, Juan Lupiáñez

**Affiliations:** ^1^Department of Organizational Behavior, University of Lausanne, Lausanne, Switzerland; ^2^Department of Social Psychology, Mind, Brain and Behavior Research Center, University of Granada, Granada, Spain; ^3^Department of Experimental Psychology, Mind, Brain and Behavior Research Center, University of Granada, Granada, Spain

**Keywords:** trust game, categorization, individuation, ingroup–outgroup perception, cooperation

## Abstract

This study investigates whether participants use categorical or individual knowledge about others in order to make cooperative decisions in an adaptation of the trust game paradigm. Concretely, participants had to choose whether to cooperate or not with black and white unknown partners as a function of expected partners’ reciprocity rates. Reciprocity rates were manipulated by associating three out of four members of an ethnic group (blacks or whites consistent members) with high (or low) reciprocity rates, while the remaining member of the ethnic group is associated with the reciprocity of the other ethnic group (inconsistent member). Results show opposite performance’s patterns for white and black partners. Participants seemed to categorize white partners, by making the same cooperation decision with all the partners, that is, they cooperated equally with consistent and inconsistent white partners. However, this effect was not found for black partners, suggesting a tendency to individuate them. Results are discussed in light of the implications of these categorization-individuation processes for intergroup relations and cooperative economic behavior.

## Introduction

Every day we come across many people. The amount of information that we can extract from these encounters can be so demanding that it needs to be organized in order to be used for making efficient decisions and plan our subsequent actions toward those people. Such organization of the information provides us with general knowledge about the individuals we intend to interact with. At the same time, this knowledge helps us guiding our interactions even with strangers.

When perceiving a person for the first time we may categorize him/her according to the salient features of his/her face such as sex, age, or ethnicity (Devine, 1989; Fiske and Neuberg, 1990). Research shows that people use stereotypes to attribute characteristics to others and consequently the impressions we form about them can be biased by those stereotypes. Interestingly, this process can take place outside the individual’s awareness (Bargh and Williams, 2006; Cunningham and Zelazo, 2007).

Social perception may involve a decision-making process where social agents decide whom to interact with and how. Perceivers try to predict the course of the interaction and whether the goals of the interaction will be achieved or not. In social contexts, this decision-making process is influenced by certain salient features of the people we interact with, such as facial expressions (e.g., Scharlemann et al., 2001; Ruz and Tudela, 2011), physical attractiveness (e.g., Solnick and Schweitzer, 1999; Solnick, 2001), or ethnicity (e.g., Sommers, 2006, 2007), which may influence our beliefs and expectations about those with whom we have to interact (e.g., Ruz et al., 2011; Gaertig et al., 2012), especially when we know nothing about them. One of the most crucial features when interacting with others concerns the level of trust deployed in these relationships. Trust is essential for a secure and healthy social life (Dunning et al., 2014), being considered as a core social motive (Fiske, 2003).

Although essential to social life, trust is conceived as irrational by philosophers (e.g., Hobbes, 1997; Machiavelli, 1515/2003) or neoclassical economists (e.g., Berg et al., 1995; Bolle, 1998). Despite that, empirical evidence has shown that people trust strangers and reward that trust (for reviews, see Johnson and Mislin, 2011; Balliet and Van Lange, 2013).

Outside the lab, trust is present in interpersonal situations (trusting a confidant), in economic markets (trusting a financial advisor), or even in political elections (trusting a government). Knowing whom to trust is crucial for preventing being deceived by others, being taken advantage of, or avoiding financial losses, and many other undesirable outcomes. When we have previous experience with our partner, the object of our trust, we can predict at different levels of certainty whether we can trust him/her, and in fact minimal interactions can already influence trustworthiness judgments (Frank et al., 1993). However, when we lack this previous experience with somebody it is difficult (although not impossible) to predict his/her behavior and consequently trust or not him/her^1^. However, trust at zero acquaintance has to be influenced by factors different from the experience with the trustee (Dunning et al., 2014).

Some research has focused on some of these factors that may influence participants trusting behavior. Among these, there is an emerging literature pointing to the role of shared group membership in the promotion of trust (Platow et al., 2012). Much of this work follow the theoretical claim by Brewer (1981, p. 356) that group membership “serves as a rule for defining the boundaries of low-risk interpersonal trust that bypasses the need for personal knowledge and the costs of negotiating reciprocity.” This proposition has been supported by the results of some studies showing that participants trust others as a function of their group membership (e.g., Tanis and Postmes, 2005), although it is not supported by results from other studies, as we will describe later (Tortosa et al., 2013).

As in many other impression formation processes, when deciding to trust unfamiliar others, we can either: (a) categorize them and interact with them according to the inferences that can be extracted from what we know (or have learnt) about their category (i.e., their inferred group membership), or (b) to individuate them and try to predict their behavior in order to know how to interact with them based solely on what we specifically learnt about them. Literature has repeatedly shown that categorization seem to be the default process in particular for social stimuli (Brewer, 1988; Fiske and Neuberg, 1990; Kawakami et al., 1998; Cuddy et al., 2004; Nelson, 2005).

In the present study we were interested in evaluating whether people infer information (i.e., reciprocation rate) about others based primarily on their category membership (i.e., ethnicity) or on individuation perception, and consequentially their decision to trust them (e.g., sharing money) depends on these reciprocation inferences. In order to do so, we adapted a procedure developed to investigate the use of social categories for the allocation of attentional control (Canadas et al., 2013), to investigate the categorization-individuation processes underlying the cooperation dynamics in a trust game context.

Canadas et al. (2013) procedure presented photographs of men and women as the context in which congruent or incongruent stimuli appeared for participants to solve a flanker task. Three faces in each social category (i.e., consistent faces) were associated either with a high proportion of congruent trials (75% congruent–25% incongruent, i.e., a low conflict context) or a low proportion of congruent trials (25% congruent–75% incongruent, i.e., a high conflict context). Whereas a forth face in each group (i.e., inconsistent face) was associated to the proportion congruent of the other group. The extent to which inconsistent faces produced the same pattern of results as consistent ones, in spite of being associated to the opposite proportion of congruency as the social category they belonged to, was taken as an index of social categorization. A categorization pattern was observed in fact in the first study, thus supporting the abovementioned idea that categorization processes seem to be the default for social stimuli.

In a second study, we manipulated the instructions given to participants, either to individuate (i.e., pay attention to the individual characteristics of the faces) or categorize (i.e., pay attention to the category-based features of the faces). The previous pattern of results was replicated in the categorization instructions group. However, the pattern of results in the individuation instructions group showed that different effects were observed for consistent and inconsistent faces, thus reflecting the individual association rather than the group associations between faces and the proportion of congruency. This pattern was taken as evidence for individuation even in social contexts when participants are motivated to do so.

In conclusion, although categorization seems to play a dominant role in person perception processing, a wide range of variables has been shown to function as modulators of categorical thinking activation, including instructions, motivation, goals, and strategies (e.g., Lepore and Brown, 1997; Castelli et al., 2004; Macrae and Cloutier, 2009); the paper by Canadas et al. (2013) showed to be a suitable method to investigate these processes. In the current study we aimed at extending this procedure to investigate individuation-categorization processes in a more direct and clear social behavior, the decisions about to trust or not a partner in an economic game.

Wilson and Kayatani (1968) examined the effect of a partner’s ethnicity on cooperation behavior in a prisoner’s dilemma game. They found that participants were far more cooperative with ingroup partners than with outgroup partners. Also, Chen et al. (2010) performed several experiments using the prisoner’s dilemma task in which participants identify themselves as members of the same university of the same ethnicity, and showed the same pattern of results as Wilson and Kayatani (1968), that is, the sense of belonging to the same group played an important role in the participants’ cooperation ratings. However, Tortosa et al. (2013) did not find evidence for this bias against the outgroup with the well-known Trust Game paradigm developed by Berg et al. (1995), originally constructed by Camerer and Weigelt (1988). In a first experiment Tortosa et al. (2013) observed no effect of ethnicity on the cooperation rate, whereas in the second experiment they observed in fact a smaller but reliable tendency toward a larger cooperation rate for the outgroup partners (64.4%) compared to the ingroup ones (57.5%).

In the current research we used a modified version of this trust game procedure and incorporated the Canadas et al.’s (2013) manipulation to investigate categorization and individuation processes. We evaluated whether participants prefer to trust (cooperate/share money with) ingroup members (i.e., white partners), compared to outgroup members (black partners). The task typically involves two players, a trustor and a trustee. The trustor (participant) is endowed with a sum of money and has to decide whether or not to share it with her/his game partner. If s/he decides to keep the money for her/himself, the trustee gets nothing. If s/he decides to share the money, the trustee receives the initial endowment multiplied by an amount (determined by the experimenter). If the trustee then reciprocates, the sum is divided between the two players; otherwise the trustor obtains nothing. In this game, the typical decision of the trustor is hazardous because the trustee’s reciprocation is not enforced by the rules. Still, substantial amounts of trust are observed across studies (Berg et al., 1995). These effects are attributed more to “social preferences” such as fairness, altruism, and reciprocation (see, for example; Fehr and Schmidt, 1999; Charness and Rabin, 2002; Fehr and Fischbacher, 2002, 2003; Fehr and Camerer, 2007) than to self-interest rational choices.

Importantly in our adaptation of the procedure, each participant was presented with two categories of faces (i.e., blacks and whites) of supposed partners randomly assigned to a high (75%) or low (25%) proportion of reciprocation in a within subject design. Also, as in Canadas et al. (2013) we manipulated that one individual in each group (inconsistent member) is associated with the proportion of reciprocation of the other group. This will allow us to examine different effects of impression formation (participant’s cooperation bias). Another advantage of using a within subject design is that it allows us to explore the learning processes underlying participants’ strategy to adapt their sharing behavior with high reciprocation vs. low reciprocation partners.

A second and more important aim of our study is to evaluate whether participants individuate, or rather categorize, that is, the extent to which participant behave in the same way with all category members, irrespectively of whether they show a consistent or inconsistent cooperation rate with rest of the category members. In case of categorization, the same decision (e.g., to cooperate with the members of one ethnic group) will be displayed also with inconsistent members of the group (that is, even with those whose reciprocation rates are opposite to the one of their own ethnic-category, and equal to the other ethnic-category). On the contrary, participants will individuate to the extent that their decisions are taken accordingly to the reciprocation rate associated to each individual face rather than to the ethnic-category. Therefore, in case of individuation, inconsistent individuals will be show different cooperation patterns than consistent faces.

We expected that along the block of trials, participants would use the facial features to categorize individuals according to the more salient features of their faces (i.e., ethnic features) and therefore decide to cooperate or not with them depending on the likelihood of their group to reciprocate. Thus, participants would share in greater extent with the individuals of the group more likely to reciprocate. However, we expected this to happen mainly for consistent individuals.

A different prediction was made for inconsistent individuals. On the one hand, and according to our previous research by Canadas et al. (2013), inconsistent individuals might be also categorized. However, given the nature of the task (Trust Game round) when participants interact several times with the same partner, previous interaction with the same person influences the participant’s decision to cooperate (King-Casas et al., 2005). Foregoing research has demonstrated that people attempting to maximize their benefits should learn from the feedback displayed after the interactions with the environment (reinforcement learning—Sutton and Barto, 1998) and consequently in our study individuation is a more efficient strategy. This strategy then should be learned quickly after the feedback of each interaction (Axelrod and Hamilton, 1981).

Taken all together, both the individualistic nature of this task and the explicit consequences of each decision (participants were informed about whether the partner reciprocated or not in each trial), we expected that participant would pay attention to each individual and therefore would individuate inconsistent partners, updating first impressions based on previous interactions (Chang et al., 2010; Campellone and Kring, 2013). This individuation pattern (i.e., a correlation between the participants cooperation rate and the individual reciprocation rate, nor the group reciprocation rate) was expected nevertheless mainly for the ingroup individuals (i.e., white partners), thus supporting previous knowledge on outgroup vs. ingroup social categorization (Judd and Park, 1988; Linville et al., 1989; Levin, 1996, 2000; MacLin and Malpass, 2001).

## Materials and Methods

### Participants

Twenty-six undergraduate white students from the University of Granada (one man, mean age 20.15 years, SD = 1.93) participated in exchange for course credits. The study was conformed to the relevant regulatory standards approved by the local ethics committee of the University of Granada in the Department of Experimental Psychology. Participants signed consent forms and received 1% of the final payoffs (maximum 10 euros).

### Stimuli and Procedure

At the beginning of the session, participants were instructed that the experiment explored the cooperation patterns that emerge between people during the so-called *trust game*. During the task participants played the role of “*trustors*.” They received 1 euro and had to decide whether to keep or share it with an allegedly partner (i.e., the “*trustee*”), an unknown person for the participant from whom a picture is shown. Each trail starts with the Euro’s symbol (€)—indicating that he/she receives 1 euro, and the participant has to decide whether to keep it (by pressing the *0* on the keyboard) or share it with their partner (by pressing *1* on the keyboard). Deciding to keep the money would yield no earnings for the partner and would end the trial. If participants decide to share, it would result in 5 euros given to the *trustee*, who, in turn, would decide whether: (a) to reciprocate the cooperation, and each of them would receive 2.5€, or (b) not to reciprocate, and the *trustee* would keep the 5€ but the participant would receive nothing. This feedback about the *trustee* decision was displayed on the computer screen 500 ms after the participant took the decision and the trial ended after the feedback. The participants’ goal was to maximize their payoffs in the game.

Participants were also informed that they were not playing with real people but that the reciprocity behavior would mimic common patterns of play by real people. Participants were not informed about the different manipulations included in the design: the ethnicity of the interaction partners or the partner’s reciprocation rate. Therefore they were unaware of the main goal of the study, which was to explore how the ethnicity of the partners can influence strategies of cooperation, and to investigate whether participants categorize vs. individuate the outgroup vs. the ingroup trustees.

**FIGURE 1:**
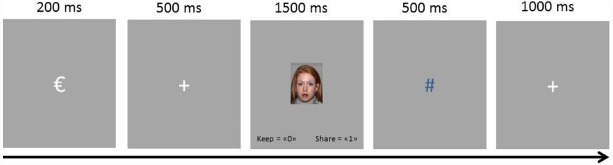
**Illustration of the procedure used during the trust game task**.

The general procedure was similar to that used by Tortosa et al. (2013). The task was presented on a PC running E-prime software (Schneider et al., 2002). Stimuli were frontal photographs of eight black people (four men and four women) and eight white people (four men–four women) from Nimstim face stimulus set (Tottenham et al., 2009) that represented the *trustees*. Faces were matched on attractiveness and trustworthiness as reported by 28 independent participants (10 men and 18 women, all whites; mean aged 32.68, SD = 6.56) in an online questionnaire using Qualtrics^®^^2^. All stimuli were presented against a gray background (see Figure 1). Each trial started with a 200 ms presentation of “€” (2.1 × 1.6° visual angle) to indicate the money given to the participant, that was replaced by a fixation point (+, 0.7 × 0.7° visual angle) for 500 ms, and was followed by the picture of the *trustee* for that trial (6.2 × 8.3° visual angle) for 1500 ms. During this time, participants had to indicate whether to keep (by pressing the “0” key) or share (by pressing the “1” key) the euro. After participants informed of their decision (or after 1500 ms in case they did not so), the picture was replaced by the fixation point for 500 ms and then replaced by a symbolic feedback symbol (1.0° × 1.0° visual angle) which indicated the *trustee*’s decision for that trial. Three possible symbols displayed in three different colors were used as feedback: a green “o”, a navy “#”, and a maroon “*”. Their meanings were: “You have decided to keep the money. You receive 1 euro. Your partner receives 0 euro”; “You have decided to share and your partner has decided to reciprocate”; “You have decided to share and your partner has decided not to reciprocate.” The association between specific symbols, color, and their meaning was counterbalanced across participants^3^. On trials where participants did not enter their decision on time (1.5 s), they saw the message “¡tarde!” (late!). At the end of the trial a larger fixation point (a “+” sign, 1.0° × 1.0°) remained on the screen for 1000 ms.

Participant played a multi-round design, with 16 different *trustees* over the course of the task. Participants played this game 40 times with each of the 16 *trustees* (for a total of 640 trials) divided in two phases of five blocks each. Each phase was designed so that three faces of an ethnic group were associated with a high probability of reciprocation rate (75%) while three faces of the other group were associated with a low probability of reciprocation rate (25%). These were consistent faces. The forth face of each group in each phase reciprocated at the rate of the other group. These were inconsistent faces. In the second phase the group reciprocation rate was inversed using four different faces for each ethnic group. The order in which black or white started reciprocating in 75% of the trials was counterbalanced across participants. Which face of the group acted as inconsistent face was also counterbalanced across participants. For instance, for a given participant, five blocks constituted the first phase. In block 1 reciprocation rate was allocated at 50% for every face of both groups, whereas in blocks 2–5 the reciprocation rate was set at 75% for three black trustees (consistent faces), and 25% for one black trustee (inconsistent face), and 25% for three white trustees (consistent faces) and 75% for one white trustee (inconsistent face). In a second phase of five extra blocks, eight new faces were presented and the reciprocation rates were inversed for the ethnic groups. That is, in the sixth block the reciprocation rate would be again set at 50% for both groups, but in blocks 7–10, three white faces would reciprocate at a 75% rate while three black faces would reciprocate at a 25% rate and one white face would reciprocate at a 25% rate while one black face would reciprocate at a 75% rate.

Once the participants finished the trust game task they were presented with the 16 faces and were asked to evaluate them using a likert-scale ranging from 1 “not at all” to 7 “very much” in what extent they were attractive and trustworthy. We also asked participants to indicate how distinctive the face was compared to the other members of its group, using a likert-scale ranging from –3 (very distinctive) to +3 (very undistinctive), and how frequently each face was presented compared to the others (1 “less,” 2 “the same,” 3 “more”). We also included some general questions about the group level, including % of reciprocation and % presentation of whites and blacks.

## Results

We analyzed the proportion of participants’ sharing/cooperation rates across conditions. First, we compared cooperation rate toward black and white trustees in the first block of the first phase (where there was no manipulation of group reciprocation rate, 50%). There was no significant difference in participants cooperation with black (mean = 0.68; SD = 0.16) vs. white trustees (mean = 0.63; SD = 0.19), *t*(25) = 1.45, *p* = 0.16.

In order to measure the categorization or individuation strategies in participants’ cooperation behavior, we analyzed it separately for each ethnic group and faces’ level of consistency (consistent or inconsistent with their respective category). Thus, cooperation rates were introduced into an ANOVA with ethnicity (black, white), block (2–5), group reciprocation rate (25%, 75%), and face consistency (consistent, inconsistent) as within-subject factors. Result showed that participants, contrary to the social categorization hypothesis, decided to cooperate equally independently of the trustee’s ethnicity, *F*(1,25) = 0.69, *p* = 0.41, ${\text{η}}_{p}^{2}$ = 0.03, that is, they did not cooperate with white trustees more than with black trustees. The main effect of Face consistency was neither significant, *F*(1,25) = 0.02, *p* = 0.90, ${\text{η}}_{p}^{2}$ = 0.00.

However, and according to our predictions, participants significantly preferred to cooperate with the group associated to high reciprocity (*M* = 66.7%, CI: 61.4–72.0) as compared to the one associated to low reciprocity (*M* = 61.9%, CI: 56.0–67.9), *F*(1,25) = 13.39, *p* < 0.001, ${\text{η}}_{p}^{2}$ = 0.35. This effect of Group Reciprocation rate was significantly moderated by block and Face Consistency, as shown by the three-way interaction between these three factors, *F*(1,25) = 4.23, *p* = 0.008, ${\text{η}}_{p}^{2}$ = 0.15. The interaction showed that the effect of group reciprocation rate (which was opposite for inconsistent faces) increased across blocks, as learning increased. This makes evident the reinforcement learning hypothesis (Chang et al., 2010) by which participants update their previous impressions with the acquired knowledge of reciprocity rate of each face.

**FIGURE 2:**
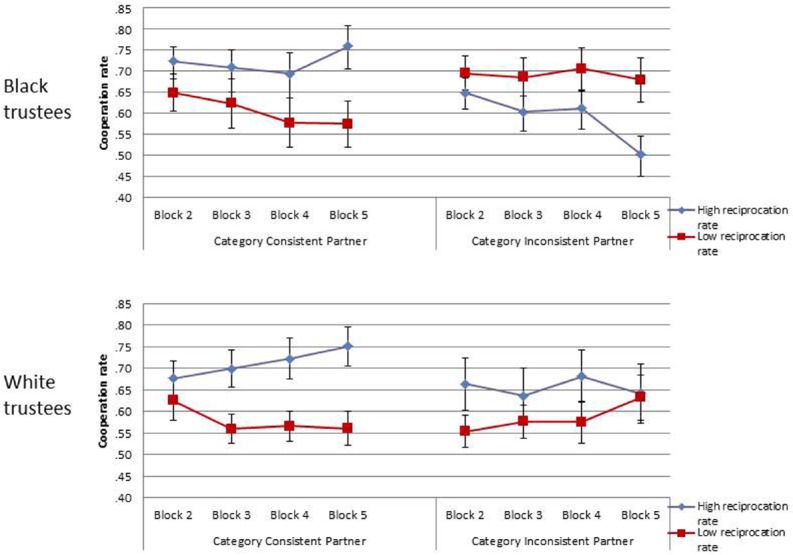
**Means of participants’ cooperation rate toward black and white trustees for each group and face consistency’ level of reciprocation**.

More interestingly, the Ethnicity by Group reciprocation rate interaction was significant, *F*(1,25) = 5.17, *p* = 0.03, ${\text{η}}_{p}^{2}$ = 0.17, and was significantly moderated by the Ethnicity × Group Reciprocation × Face consistency three-way interaction, which was also significant, *F*(1,25) = 10.47, *p* = 0.003, ${\text{η}}_{p}^{2}$ = 0.30. Importantly, contrary to our predictions, a significant Group Reciprocation rate by Face Consistency interaction was observed for black trustees, *F*(1,25) = 6.92, *p* = 0.01, ${\text{η}}_{p}^{2}$ = 0.22, while the same interaction was not significant for white trustees, *F*(1,25) = 0.70, *p* = 0.41, ${\text{η}}_{p}^{2}$ = 0.03 (see Figure 2). That is, while black trustees led to cooperation responses as a function of the faces’ individual cooperation rates (as they were opposite for inconsistent faces), in the case of the white trustees the participant’s cooperation behavior was guided by the cooperation rate of the group, independently of the individual cooperation rate (i.e., independently of face consistency). As the same cooperation responses for consistent and inconsistent faces can be conceived as a sign of *categorization*, and opposite cooperation behaviors for consistent vs. inconsistent faces as a sign of *individuation*, these interactions indicated that black trustees were individuated whereas white trustees were categorized.

### Trustees’ Evaluations

We checked for individual differences of the faces. Specifically, first we wanted to evaluate how the trust game task could have affected judgments of attractiveness and trustworthiness of the trustees. We then performed a repeated measure analysis (two group reciprocation rate by two ethnicity by two face consistency) on each dependent variable. We did not find any significant effect nor interaction for attractiveness, *Fs*(1,25) < 2.8, *ps* > 0.11. Trustworthiness ratings only revealed a significant interaction effect for Ethnicity by Face Consistency, *F*(25) = 4.57, *p* = 0.04, ${\text{η}}_{p}^{2}$ = 0.16, indicating that consistent black trustees were evaluated as more trustworthy (mean = 3.9; SD = 1.61) than inconsistent black trustees (mean = 3.5; SD = 1.53). However, inconsistent white trustees were evaluated as more trustworthy (mean = 3.7; SD = 1.10) than consistent white trustees (mean = 3.4; SD = 1.32). All other *Fs*(1,25) < 2.5, *ps* > 0.14.

Then we analyzed how distinctive (very distinctive –3 to very undistinctive +3) each individual faces was in comparison with the ingroup faces. The only significant effect was the Group Reciprocation rate by Ethnicity interaction, *F*(25) = 4.24, *p* = 0.05, ${\text{η}}_{p}^{2}$ = 0.15. The result showed that black trustee associated with low reciprocation rates were perceived as more similar to each other (mean = 1.07; SD = 1.47) than those associated to high reciprocation rates (mean = 0.89; SD = 1.41) while white trustees were perceived as more similar to each other when associated to high group reciprocation rates (mean = 0.96; SD = 1.46) compared to low group reciprocation rates (mean = 0.67; SD = 1.62). None of the other effects reached significance, *Fs*(1,25) < 1, *ps* > 0.35. Next we perform the same analysis to evaluate the perception of the frequency of individual faces presentation (1 “less,” 2 “the same,” 3 “more” compared to the rest). None of the effects were significant, *Fs*(1,25) < 1.5, *ps* > 0.22.

We next evaluated how participants perceived the faces at a group level. We first analyzed how frequently participant believed that the two groups of faces (blacks and whites) were presented during the task. There was no significant differences in their estimates of the overall presentation rates of black trustees (mean = 56.15%; SD = 12.11%) compared to white trustees (mean = 51.92%; SD = 15.43), *t*(25) = 1.10, *p* = 0.28, ${\text{η}}_{p}^{2}$ = 0.01. This result indicates that participants correctly estimated that all faces were equally presented throughout the experiment. Then we evaluated participants’ impression about the reciprocation rates of black and white trustees. Interestingly, we found a significant difference in overall reciprocation’ judgments depending on the ethnicity of the trustee, *t*(25) = 2.95, *p* = 0.005, ${\text{η}}_{p}^{2}$ = 0.26. Participants reported they thought that black trustees reciprocated more often (mean = 60.58%; SD = 13.44) than white trustees (mean = 48.50%; SD = 16.00).

## Discussion

The present study explored the effect of ethnicity and consistent vs. inconsistent behaviors (reciprocation rates) regarding their identity group in a multi-round trust game task. We wanted to explore whether ethnicity moderates the decision of whether to cooperate with partners or not and, more importantly, whether social categorization or individuation processes would underlie those decisions. Results revealed that participants did not show a particular bias toward cooperating with white compared to black in general, although, interestingly, they used different strategies to make decisions about how to cooperate (share money) or not with white and black partners. Whereas the observed pattern of results led us to conclude that the white ingroup trustee’s faces were categorized (i.e., the same cooperation pattern was observed for consistent and inconsistent faces), the black outgroup trustee’s faces were individuated (i.e., an opposite pattern of cooperation was observed for consistent and inconsistent faces).

Even though preferences to cooperate with ingroup members more than with outgroup members have been largely reported in previous research (Wilson and Kayatani, 1968; Tanis and Postmes, 2005; Chang et al., 2010), other studies’ results go in opposite direction, that is, favoring outgroup members (see Allport, 1954; Monteith et al., 2002; Tortosa et al., 2013, study 2). However, our results did not show any bias neither for black nor for white trustees as measured in block 1 (50% reciprocation rate for both black and white trustees). This finding is in line with previous results by Stanley et al. (2011) which show that unless participants had a strong pro-whites or pro-black bias, as measure with an implicit ethnic attitudes test, their evaluation of trustworthiness and their cooperation behavior (economic offers in a trust game) kept similar toward black and white partners.

We can rule out the possibility that black and white trustees evoked different trustworthiness impression, as we controlled for this (among other variables, e.g., attractiveness) with the pretest for stimuli selection. The evaluation of the trustworthiness of the stimuli at the end of the trust game did not show either overall differences between black and white trustees, which go in line with the pretest and with other studies investigating ethnic attitudes (Phelps et al., 2000; Stanley et al., 2011).

A potential explanation for the similar cooperation toward partners belonging to both ethnic groups can be due to the use of women and men as stimuli. It may exist a confound between these two groups, so participants prefer to cooperate with women more as they are perceived more trustworthy than men (independently of their ethnic categorization, Buchan et al., 2008) and consequently the gender bias may have concealed the ethnic bias. This is surely a confound factor that should be carefully analyzed by future research.

Interestingly, the manipulation of consistency significantly affected the evaluation of trustworthiness, which may explain the current results in our study. The different evaluation of inconsistent black and white faces being the former more positively evaluated regarding trustworthiness than the later may evidence that people accepted more ingroup members (whites) that behave unexpectedly compared to outgroup members (blacks; Kosic et al., 2014).

Our main contribution to the study of ethnic categories and decision-making literature focuses on the study of cooperation strategies related to categorization and individuation processes. Result showed that (white) participants used different strategies to make decisions on how to cooperate (share money) with white and black partners. Specially, they learnt which face is behaving inconsistently with the rest of the group and decided how to cooperate with this person accordingly to the specific cooperation rate that he or she showed. That is, participants individuate each trustee they were encountering with. Interestingly, however, this individuation strategy applied exclusively to black faces (outgroup members). Contrary, decisions to cooperate toward white trustees followed a categorization strategy. That is, participants took their decisions to cooperate with inconsistent trustees as a function of the proportion of reciprocation assigned to the majority of the white trustees (consistent trustees).

The reason why participants categorize whites and individuate blacks, contrary to our expectations, and to what was previously shown (Hugenberg et al., 2010) is far from being clear. However, it could be argued that participants may care about ingroup identification (Castano and Yzerbyt, 1998); therefore, they may be motivated to preserve the homogeneity of the ingroup members (Castano, 1999) producing the categorization effect observed for white faces. Furthermore, according to interdependence theories, participants may have individuated black faces given that their outcomes (the money they could earn during the task) may depend on their sharing behaviors (Ruscher and Fiske, 1990). Participants may have paid special attention to black people to compensate their dispositional behavior to categorize them and by consequence they increased their attention to inconsistencies among black partners. This increased attention may have helped them to use the strategy to cooperate with each face according to the individual reciprocation rate rather than the group reciprocation rate.

Another alternative explanation comes from Collins et al. (2011) model of learning phenomena, and concretely the “blocking” (Kamin, 1968) explanation, explaining why people learnt with different strategies about black and white partners. “Blocking” might occurs for whites when a new proportion of reciprocity (inconsistent-cue) is introduced alongside a proportion of reciprocity (consistent-cue) whose meaning has already been learned about the majority of the members of the group. Because the perceptual information coming from the inconsistent partner (white person) is redundant at the perceptual level (providing no additional information beyond the original cue), learning about it may have been blocked.

Interestingly, while blocking could explain the null effect (more related to categorization for whites), highlighting could explain the individuation effect for blacks. Highlighting occurs when a person focuses extra attention on a cue that changes the meaning of a previously learned cue, as happens when a learned association is no longer correct when a new cue is added alongside a known one (Kruschke, 2009). Another explanation to blocking from a motivational perspective will indicate that for white participants it is not enriching on a matter of novelty to learn about others whites, but it is highly interesting to know about the outgroup, to avoid threats (highlighting).

Unfortunately we do not have information about participants’ previous experience with black individuals, so future studies should measure and control for it. Future research should also focus in explaining the mechanism underlying the individuation—categorization strategies chosen by the participants, not only in economic games, but also in other social interactions, such as prosocial behaviors. It will be also interesting to know whether bottom-up (perceptual information) or top-down (conceptual-stereotypes) processes influence judgmental tasks. Previous research in gender-emotion stereotypes (Becker et al., 2007) show that both top-down and bottom-up processes can co-occur during people evaluation.

Another specific detail of our procedure is that another group category apart from the ethnical group (i.e., gender) could be salient, as half of the faces in each group were women whereas the other half were men. Given that the majority of participants in our study (all but one) were women this might have affected the pattern of results. However, given that this occurred for the ethnic groups it seems unlikely that it could explain the pattern of results. Nevertheless, future research should control more carefully for the presence/absence of different important category features (ethnicity, gender, age, etc.).

It seems clear that future research is necessary to replicate and consolidate the specific findings observed in the reported study, and to better explain the observed pattern of results. Nevertheless, and importantly, the current study has shown to be a suitable tool to investigate the incidental generation and use of categorization-individuation social cooperation processes. In a previous study (Canadas et al., 2013), this general paradigm showed to be also suitable to investigate these categorization-individuation processes and their use underlying the implicit allocation of attentional control. We believe this paradigm could be extended to the study of other situations where categorization vs. individuation processes play an important role in social interactions. Perhaps the individuation pattern observed for the outgroup members might disappear whenever more than four members from each category have to be tracked. Therefore, our procedure might be useful to investigate the interplay between using specific knowledge about our interaction with a particular individual to predict his/her future behavior vs. using knowledge we have about our previous interactions with other members of the same group, and the boundary conditions for the use of one process or the other.

### Conflict of Interest Statement

The authors declare that the research was conducted in the absence of any commercial or financial relationships that could be construed as a potential conflict of interest.

## Footnotes

1 Trusting behavior is the willingness of an individual to expose themselves to the actions of others while trustworthy behavior is defined to be rewarding trust through reciprocation.

2 There was neither a significant difference in attractiveness between the two ethnic faces *t*(27) = 1.64, *p* = 0.11; *Alpha*_foralltheblackfaces_attractiveness_ = 0.79, mean = 2.67, SD = 0.61; *Alpha*_forallwhitefaces_attractiveness_ = 0.81; mean = 2.51, SD = 0.57; nor a significant difference in trustworthiness *t*(27) = 0.19, *p* = 0.85; *Alpha*_foralltheblackfaces_trustworthiness_ = 0.77, mean = 2.93, SD = 0.52; *Alpha*_forallwhitefaces_trustworthiness_ = 0.71; mean = 2.91, SD = 0.50.

3 This procedure was used because in a follow up experiment we wanted to evaluate evocated potentials associated to the feedback as have been previously studied in Tortosa et al. (2013).
